# Preparation and Properties of Titanium Obtained by Spark Plasma Sintering of a Ti Powder–Fiber Mixture

**DOI:** 10.3390/ma11122510

**Published:** 2018-12-10

**Authors:** Mingjun Shi, Shifeng Liu, Qingge Wang, Xin Yang, Guangxi Zhang

**Affiliations:** 1 School of Metallurgical Engineering, Xi’an University of Architecture and Technology, Xi’an 710055, China; mj_hour@163.com (M.S.); webdymewqg@163.com (Q.W.); ZGX15502979951@163.com (G.Z.); 2 College of Materials Science and Engineering, Xi’an University of Technology, Xi’an 710048, China; yangx@xaut.edu.cn

**Keywords:** spark plasma sintering, porous titanium, titanium fiber, titanium powder, composite pore structure

## Abstract

Porous titanium is a functional structural material with certain porosity, which is prepared from titanium powder and titanium fiber. In order to study the porosity, phase structure, microstructure, sintering mechanism and mechanical properties of porous titanium obtained by spark plasma sintering of a Ti powder–fiber mixture at different sintering temperatures, a spherical titanium powder (D_50_ of 160 μm) was prepared via plasma rotating electrode processing, and titanium fiber (average wire diameter of fiber of 110 μm) was prepared by drawing, and they were mixed as raw materials according to different mass ratios. Porous titanium with a fiber–powder composite porous structure was prepared by spark plasma sintering at sintering temperatures of 800 °C, 900 °C and 1000 °C under a sintering pressure of 20 MPa. The results showed that there were no new phases occurring in porous titanium with porosity of 1.24–24.6% after sintering. Titanium fiber and titanium powder were sintered using powder/powder, powder/fiber and fiber/fiber regimes to form composite pore structures. The mass transfer mechanism of the sintered neck was a diffusion-dominated material migration mechanism during sintering. At higher sintering temperatures, the grain size was larger, and the fiber (800 °C; 10–20 μm) was finer than the powder (800 °C; 10–92 μm). The stress–strain curve of porous titanium showed no obvious yield point, and the compressive strength was higher at higher sintering temperatures. The results of this paper can provide data reference for the preparation of porous titanium obtained by spark plasma sintering of a Ti powder–fiber mixture.

## 1. Introduction

Porous titanium is a structural and functional integrated material with useful properties that are inherited not only from the physical and chemical properties of Ti, such as wear resistance, corrosion resistance, high specific strength and good biocompatibility, but also specific functional properties that are imparted to the material due to its porous structure, including low density, large specific surface area, low weight and good permeability. Porous titanium is widely used in biology, medicine, aerospace and other industries [1,2,3,4,5]. The manufacturing methods of traditional metal porous materials include casting, slurry foaming, powder metallurgy and others. Most porous titanium materials produced by these methods have some shortcomings, such as coarse grains, uneven distribution of pores, stress concentration of pores, dispersive mechanical properties, easy oxidation and so on [6,7,8]. Spark plasma sintering (SPS) is a low-temperature, fast, environmentally sustainable powder sintering technology. This technology integrates plasma activation, hot pressing and resistance heating, and directly connects the high-frequency pulse current between the pressurized powder particles. This technology depends upon heat from the plasma produced by spark discharge, which has the advantages of fast heating and cooling speeds, a low sintering temperature, short sintering time, fine and uniform grain size, controllable microstructure, and so on [9,10,11]. Therefore, advantages of SPS technology can be used to try to solve the defects of traditional porous titanium.

Yang Xin et al. [12] used plasma rotating electrode processing (PREP) spherical Ti-45Al-2Cr-2Nb-0.2W prealloyed powder as raw material, and successfully prepared a TiAl-based composite material with a relative density of 99.2% by SPS technology. The results show that with an increase in the sintering temperature, the microstructure changes regularly, and two-dimensional structures, near-layer structures and full-sheet structures appear. The microstructure of the sintered body is uniform. Zhang Lei et al. [13] used SPS technology to prepare Ti-Ag/Ti porous composites with a porosity of 49% and an average pore diameter of 105 μm, which were pure and uniform in composition. Guo Yu et al. [14] prepared a titanium-based tricalcium phosphate composite by adding Ti mesh to a Ti/α-TCP mixed powder. The results show that the compressive strength of the material was enhanced by the addition of titanium mesh as a framework. The compressive strength of the Ti/α-TCP/Ti mesh composite was as high as 590 MPa at a sintering temperature of 900 °C. Currently, porous Ti is mostly prepared by powder/powder or fiber/fiber sintering, its pore structure is single, and its pore size is randomly distributed within a certain range. The porosity of samples prepared by powder is not significantly high. The sample prepared by fiber has a larger porosity but less desirable mechanical properties [15,16,17]. Besides, it was found that tritium storage materials have coarse grain and few grain boundaries in the research of tritium storage materials [18,19]. Helium produced by tritium decay mainly exists in grains and accumulates at grain boundaries. When the concentration exceeds the critical threshold, the material will fail [20,21]. It is well known that pure titanium is one of the materials with the highest hydrogen absorption density in metal materials, and the biggest advantage of SPS in the preparation of titanium alloys is the suppression of grain growth [22]. Therefore, it is a feasible method to prepare porous titanium obtained by SPS of a powder–fiber mixture. The interfacial effect of powder/fiber and the superposition effect of various composite pore structures can be used to optimize the porous Ti structure and improve the properties of porous titanium for tritium storage [23].

In this study, a PREP spherical titanium powder and titanium fiber were used as raw materials to construct a composite structure. SPS technology was used to prepare porous titanium with a composite pore structure. The phase composition, pore structure, microstructure and mechanical properties of the samples before and after sintering were analyzed and tested. The effects of the sintering temperature and sintering pressure on the sintering behavior, microstructure evolution and compressive properties of fiber/powder were studied, providing a theoretical basis for studding composite porous titanium with interfacial and pore structure superposition effects.

## 2. Materials and Methods

### 2.1. Material

**Figure 1:**
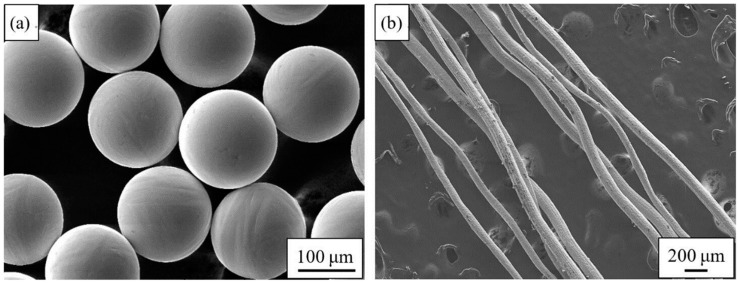
SEM images of the raw materials: (**a**) Ti powder; (**b**) Ti fiber.

The experiment uses spherical pure titanium powder (purity > 99.75%, D_50_ = 160 μm, purchased from Shaanxi Yuguang Feili Metal Materials Co., Ltd., Xi’an, China) prepared by PREP, and the titanium fibers (purity ≥ 99.60%, average diameter: 110 μm, purchased from Anping County Dakewei Metal Wiremesh Product Co., Ltd., Xi’an, China) prepared by the drawing method. According to the national standard GB/T 1482-2010-Metallic powders-Determination of flow time by means of a calibrated funnel (Hall flowmeter), Chinese Standards (Beijing, China, 2010), the apparent density and flowability of the original powder were measured by Velocity Instrument FT-102 (Ningbo Rooko Instrument Co., Zhejiang, China), and the mean value was obtained after three tests. The results show that the apparent density is 2.66 g/cm^3^ and the flowability is 28.88 s/50 g. Figure 1 is the SEM image of the raw materials. It can be seen from the Figure 1a that the PREP powder has a high sphericity, a smooth surface, and no “satellite balls”, hollow powder or other defects. The surface roughness of the fiber (Figure 1b) after etching is large, and the wire diameter is not uniform.

### 2.2. Preparation of Porous Titanium

The fiber was woven into a mesh and then it was cut into a wafer of Ф20 mm, which was etched to remove the surface oxide layer. The pickling solution was prepared at a ratio of HF:HNO_3_:H_2_O = 1:3:17, and the pickling time was 30 s. After etching, the titanium mesh was immersed in clean water for 3 min, then placed in absolute ethanol, washed in an ultrasonic cleaner for ten minutes, and then taken out, and then dried by cold air.

A graphite die having an inner diameter of Ф20 mm was filled with titanium powder and titanium fiber mesh. They were laid in three mass ratios of 1:0.08, 1:0.16 and 1:0.24. Firstly, 2 g of titanium powder was placed at the bottom of the die to prevent direct contact between graphite carbon paper and titanium fiber, resulting in uneven mixing, then a layer of titanium fiber mesh was laid in, and the corresponding quantity of titanium powder was poured into the die. Each operation was horizontally oscillated 5 times to ensure even distribution, and the process was repeated 10 times. Finally, 2 g of titanium powder was laid on the last layer of fiber mesh. The graphite die was filled and placed in a sintering furnace.

HPD25/3 SPS equipment (manufactured by FCT, Munich, Germany) was used for sintering at 800 °C, 900 °C and 1000 °C, and the sintering pressure was 20 MPa. The heating rates were: 100 °C/min from 0 °C to (T—200) °C; 10 °C/min from (T—200) °C to (T—100) °C; 5 °C/min from (T—100) °C to (T—50) °C; and 2 °C/min from (T—50) °C to T °C. After sintering to the specified temperature, heating was stopped by maintaining the pressure for 5 min, and a Ф20 mm sample was obtained by cooling to room temperature.

### 2.3. Characterization

In order to remove the carbon paper adhering to the sample surface and the carbon contamination layer caused by the graphite die, the specimens were mechanically polished after cutting. The Archimedes method was used to determine the density of the samples with distilled water as the liquid medium. The phase composition of the samples before and after sintering were characterized by X-ray diffraction (XRD, D8 Advance, Bruker, Karlsruhe, Germany) using Cu Kα irradiation at 40 kV from 30° to 90°, and the scanning step size was 0.02°. Scanning electron microscopy (SEM, Philips XL30, Amsterdam, Holland) and optical metallographic microscope (OM, OLYMPUS GX51, Tokyo, Japan) were used to characterize the microstructures. The grain size was measured by a mean linear intercept method. The mechanical properties of specimens were characterized by compression tests on an HT-2402/100 (Hung Ta Instrument Co., Ltd., Shanghai, China) material mechanics testing machine, the compressed sample size was Ф6 × 9 mm and the testing speed was 0.5 mm/min.

## 3. Results and Discussion

### 3.1. Phase Analysis

**Figure 2:**
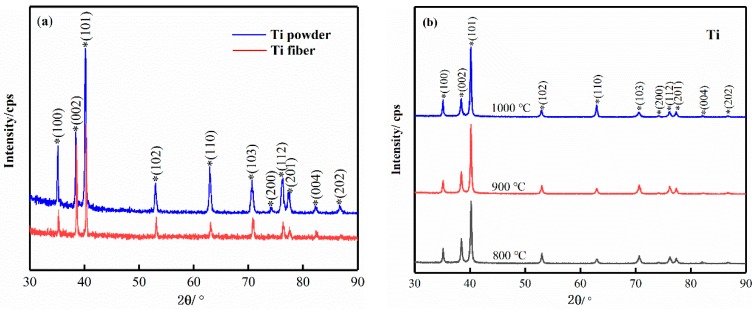
XRD patterns of raw materials and samples: (**a**) is the original Ti powder and Ti fiber; (**b**) is the samples with different sintering temperatures (800 °C, 900 °C and 1000 °C).

The composition of the sample phase before and after sintering at different sintering temperatures was analysed by XRD, and the diffraction patterns are shown in Figure 2. It shows that the raw materials and the samples sintered at different temperatures have a single-phase composition, and the main phases are all typical close-packed hexagonal α-hcp Ti phases. The diffraction peaks are similar in shape and no new ones occurred during sintering. The phase formation indicates that the sample is free of oxides, nitrides and so on during the SPS sintering process, however it is related to the detection accuracy of the XRD device. The diffraction peak intensity of titanium powder is higher than that of titanium fiber in Figure 2a, which indicates that the surface crystallinity of titanium powder is better than that of titanium fiber. The reason may be that the drawing process leads to more crystal defects of titanium fiber, which makes the surface crystallinity decrease and the peak value of XRD smaller. In Figure 2b, the XRD patterns of porous titanium prepared at different sintering temperatures are very similar. The phase composition of porous titanium is the same, and no new phase occurred. The relative intensity and width of diffraction peaks vary little within the allowable range, indicating that the sintering temperature has little effect on the phase composition of porous titanium.

### 3.2. Microstructure and Pore Characteristic

**Figure 3:**
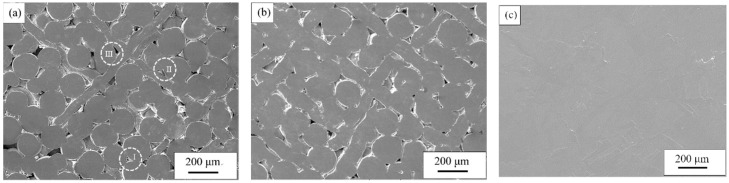
SEM micrographs of samples sintered at different sintering temperatures: (**a**) 800 °C, (**b**) 900 °C and (**c**) 1000 °C.

Figure 3 shows the SEM images for samples at different sintering temperatures. Figure 3a–c represent 800 °C, 900 °C and 1000 °C, respectively. When the sintering temperature was 800 °C (Figure 3a), the powder and fiber were uniformly mixed. After sintering, the powder/powder, powder/fiber and fiber/fiber arrangements could be metallurgically combined to form a sintered neck, and according to the different overlapping methods, the composite pores of different structures are formed. The shape factor (*F*) [24] is used to quantitatively describe the sample pore structure:

(1) $$F=\frac{4\pi A}{\rho_{\rho }^{2}}$$

where *A* is the cross-sectional area of the hole and *ρ_p_* is the circumference of the hole. The pores composed of powder/powder (Figure 3aI) are typical spherical powder sintered pore structures with an aspect ratio of 5 and a shape factor *F* of 0.72. The pores are generally large and there is a typical defect of powder sintering—the bridging effect (indicated by the arrow), and this phenomenon causes uneven density of the sample due to the poor fluidity of the powder, which is caused by the overlapping of the powders during the particle rearrangement phase. The pore structure consisting of powder/fiber is divided into two types, one consisting of powder/powder/fiber (Figure 3aII) and the other is made of powder/fiber/fiber (Figure 3aIII). The pore morphology is narrow with long slot pores. The bonding area between powder and fiber is larger than that between powder and powder because of the bending deformation of fiber during the contact between powder and fiber. Consequently, the size of the sintering neck is larger and the pore size is slightly smaller than that of the pore formed by powder/powder after sintering. The *F* of the pores composed of powder/powder/fiber tends to 0, and the pore shrinkage spheroidization process is embodied as the growth of the sintered neck composed of powder/powder and powder/fiber; the pores composed of powder/fiber/fiber have an aspect ratio of 1, and a shape factor *F* of 1.0, are small in number and relatively uniform in size, and the pore shrinkage spheroidization process is embodied by the growth of the sintered neck between the powder/fiber and the fiber/fiber. When the sintering temperature rises to 900 °C (Figure 3b), the three kinds of pores shrink, the pore size decreases, the inner wall is smooth and round, and the sintering neck grows obviously. The reason is that when the temperature rises, the atomic self-diffusion coefficient increases, and the diffusion ability increases, which accelerates surface diffusion, volume diffusion and other diffusion modes, but because of the different morphologies of the powder and fiber, there are still some macropores under random arrangement, and there are two kinds of size effects. At 1000 °C (Figure 3c), it can be seen that the density of the sample increases at this time, the powder and the fiber may have completely melted, only a few small pores exist on the surface, and the pore diameter is small and spheroidized. Closed pores become the main pores and the pore structure is difficult to resolve. Densitometry was performed on samples with different sintering temperatures using the Archimedes method, and the sample porosity was determined. As a result, when the sintering temperature was 800 °C, 900 °C and 1000 °C, the porosity was 22.91%, 7.58% and 2.25%, respectively, and the porosity of the sample showed a decrease as the sintering temperature increased. What we hoped to obtain is a porous titanium which not only has a certain porosity but also has a good pore structure and morphology. When the sintering temperature is 800 °C, the sample has a large porosity and a large pore size, and when the temperature is 1000 °C, the opposite is true. Only when the sintering temperature is 900 °C, the porosity and pore structure of the samples meet the expected results. Therefore, it is considered that with porous titanium having a composite pore structure prepared via titanium powder and titanium fiber, the optimum sintering temperature is 900 °C.

**Figure 4:**
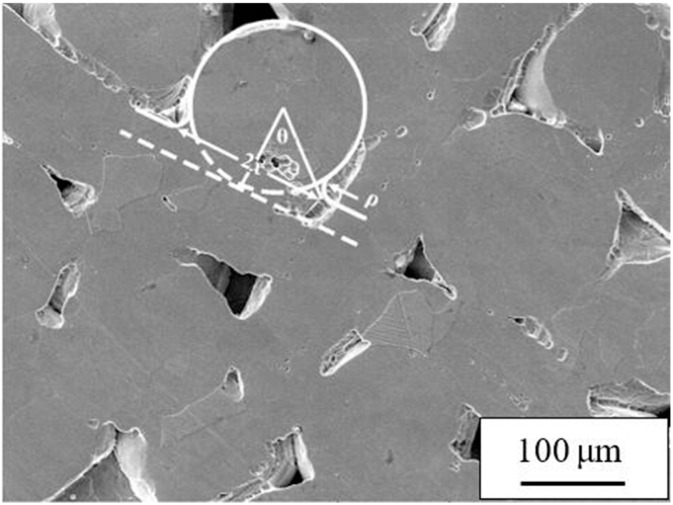
The ball-plate model on the surface of the sample: the sintering temperature is 900 °C and the sintering pressure is 20 MPa.

It is generally believed that during the SPS sintering process, the spheroidization and shrinkage of the pores, the densification of the sample, and so on are the combined effects of mechanical pressure, discharge plasma, high-intensity pulse current, and so on, and the change from quantity to quality on the particle bonding surface is caused by the different material migrations. During the sintering process, material migration can follow different paths, the main mechanisms including viscous flow, plastic flow, evaporation condensation, surface diffusion, volume diffusion and grain boundary diffusion [25,26,27]. The formation and growth of the sintered neck are the result of different material migration mechanisms. In this experiment, the fiber and the powder are mixed and sintered, and the fiber/fiber, fiber/powder and powder/powder undergo metallurgical bonding under the migration of Ti atoms to form a sintered neck. Assuming the fiber is a plate and the powder is a ball during the sintering process, when the sintering starts, the plate and the ball change from mechanical to metallurgical bonding due to atomic migration, which is in accordance with Kuczynski’s theory of the ball-cylinder configuration [28,29,30]. The kinetic equations of the length of the sintered neck under different migration mechanisms are summarized as follows:

(2) $${\left(\frac{x}{a}\right)}^{n}=\frac{F\left(T\right)}{a^{m}}t$$

where *x* is the radius of the sintered neck, *a* is the radius of the particle, *F* (*T*) is the temperature function, *t* is the sintering time, and *m* and *n* are exponents related to the migration mechanism. The derivative of the ratio of *lg*(*x/a*) to *lg*(*t*) is the sensitivity index n. Figure 4 is a local magnification diagram of the microstructure of the sample at 900 °C. The mass transfer of the sintered neck formed by powder and fiber is determined by the ball-cylinder sintering model, and finally *m =* 4, *n =* 6 was obtained, which shows that the fiber–powder composite structure material was sintered in the early stage, and that the growth of the sintered neck was governed by a mass migration mechanism dominated by grain boundary diffusion and supplemented by other diffusion mechanisms. The vacancy gradient near the neck seems to result from the concave flow to the grain boundary, resulting in the growth of the sintering neck and the smoothing and contraction of the holes. The *F* (*T*) temperature function equation related to grain boundary diffusion is as follows:

(3) $$F=\frac{96\gamma_{sv}\Omega WD_{gb}}{kT},$$

where *γ_SV_* is the free energy of solid gas surface, Ω is the Ti atom volume, *W* is the grain boundary thickness, *D_gb_* is the grain boundary diffusion coefficient, *k* is the Boltzman constant, and *T* is the thermodynamic temperature.

As the sintering time increases, the n value increases, and the main diffusion mechanism of the sintering neck length changes from grain boundary diffusion to surface diffusion, while other diffusion mechanisms become auxiliary, and vacancies flow from the concave neck surface to a granular convex surface. The reverse flow of atoms causes the neck growth and the smooth surface of the hole. The *F* (*T*) temperature function equation related to surface diffusion is as follows:

(4) $$F=\frac{56\gamma \delta^{4}D_{s}}{kT},$$

where *δ* is the lattice constant and *D_s_* is the surface diffusion coefficient. The other parameters are the same as above.

In addition, during the SPS sintering process, the intermittent release of the DC pulse voltage generates a discharge plasma in the voids of the powder and the fiber, so that the particles inside the sintered body uniformly generate Joule heat and activate the particle surface, thereby reducing the diffusion free energy of the Ti atom. At the same time, due to the effect of external axial pressure, the migration of matter is strengthened, which accelerates the formation and growth of the sintered neck, and the spheroidization and shrinkage of the pores [10,22,31].

**Figure 5:**
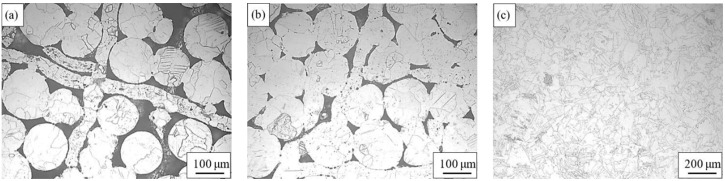
OM micrographs of samples sintered at different sintering temperatures: (**a**) 800 °C, (**b**) 900 °C and (**c**) 1000 °C.

The grain sizes of the samples produced with different process parameters were observed by OM microscope, and the grain size was measured by the intercept method. Figure 5 shows a microstructure photo of samples after different sintering temperatures. At the sintering temperature of 800 °C in Figure 5a, the microstructure of the sample was mainly composed of equiaxed α and acicular α, and contained few twins. Some of the grains formed through the sintered neck to form worm α, with an equiaxed α size of 10–92 μm. The grain size on the Ti fiber was 10–20 μm, which was finer than the Ti powder. At 900 °C (Figure 5b), the microstructure of the sample was mainly equiaxed α, and it contained a small amount of needle-like α and lamellar β. The size of the equiaxed crystal was 30–110 μm, the thickness of the lamellar β was 20–100 μm, and the length was 50–120 μm. At a sintering temperature of 1000 °C (Figure 5c), the degree of densification of the samples was high, and the microstructure was mainly composed of serrated alpha and lamellar beta tissue. The thickness of the β layer was 15–60 μm, and the length was 80–300 μm. Comparing the grain sizes at three different temperatures, we found that at higher sintering temperatures, the grain nucleation was higher, and the size was relatively uniform but was also larger at higher temperatures. Between 800 °C and 900 °C, the grain size on the fiber was significantly smaller than that on the powder, which was also smaller than the grain size (90–160 μm) of the porous material of titanium fiber prepared by our team [32]. In the process of grain growth, the effect of crystal defects on grain growth generally serves two purposes: one is to increase the internal stored energy of the material, which provides driving force for recovery and recrystallization; the other is that the defect will aggregate at the grain boundary, hindering migration and growth of grain boundary. Therefore, it can be considered that this is the reason why the grain size on the fiber is smaller than that on the powder, and this is also consistent with the previous XRD analysis that the diffraction peak value of the titanium fiber is lower than that of the titanium powder. At 1000 °C, the density of the sample is relatively high, and the fiber and powder have been alloyed together, and it is difficult to distinguish the grains from the microstructure which are located on the fiber, powder or node, so we do not focus on that analysis.

### 3.3. Mechanical Properties

**Figure 6:**
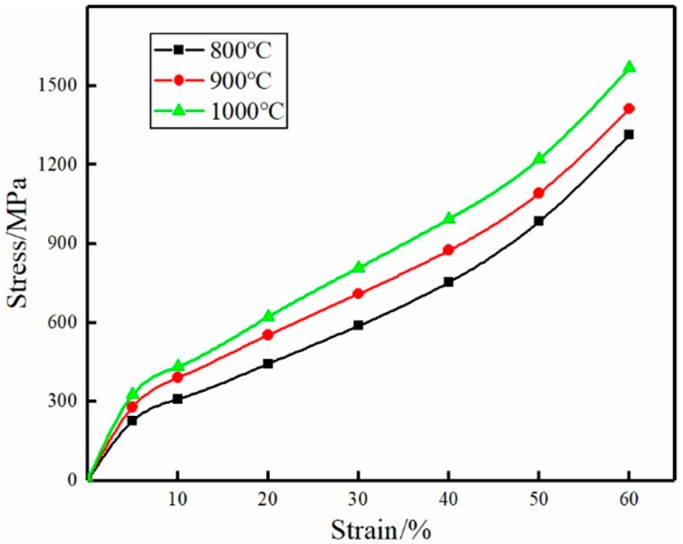
Stress–strain curves of the sample sintered at different sintering temperatures.

Figure 6 shows the static compression stress–strain curves of porous titanium at various sintering temperatures. The stress–strain curves of porous titanium can be divided into three stages: the elastic deformation zone, yield platform region and densification zone where the stress increases rapidly, which is consistent with the compression process of porous foam materials described by Gibson-Ashby [33]. The elastic area was long, approximately 5%, and it was not possible to determine whether the materials had a significant yield point. At sintering temperatures of 800 °C, 900 °C and 1000 °C, the σ_0.2_ values were 228.04 MPa, 254.49 MPa and 318.31 MPa, respectively. Under continuous pressure, porous titanium enters the yield platform, where the pore collapses and shrinks, the fiber begins to fracture after reaching the critical binding force, and the compressive strength of the sample increases slowly. At 30% strain, the compressive strengths of materials prepared at 800 °C, 900 °C and 1000 °C were 582.15 MPa, 702.54 MPa and 799.31 MPa, respectively. The compressive strength was higher with higher temperatures. In the third stage, porous titanium is densified under axial pressure, at which the pores have basically collapsed, and the strain decreases as the stress increases sharply on the stress–strain curve.

The strength of composite porous titanium depends on the fusion bonding between powder/powder, fiber/powder and fiber/fiber. At higher sintering temperatures, the activity of Ti atoms is greater and the diffusion flow between powder/fiber is elevated. Consequently, more sintering nodes are formed, enhancing the strength of the material. At the same time, the composite pore structure formed by fiber and powder, as well as the toughness of the fiber itself, will give the sample a binding force. This is why the compressive strength of porous titanium having a composite pore structure is higher than that of porous titanium prepared by using only titanium powder [34] or titanium fiber [17] as a raw material.

## 4. Conclusions

(1) After preparation of composite pore structure porous titanium with a Ti fiber–powder mixture by SPS technology, there were no new phases occurring in porous titanium with porosity of 1.24–24.6% after sintering, and the main phase of the porous titanium was α-hcp Ti;
(2) Titanium fiber and titanium powder formed a composite pore structure through powder/powder, powder/fiber and fiber/fiber sintering. The formation and growth of the sintered neck were dominated by the grain boundary diffusion mechanism and surface diffusion mechanism. The surface grain of the fiber was more uniform and finer than that of the powder, and other diffusion mechanisms were supplemented. The grain size of the sample grew with the increase of sintering temperature, and the grain surface grain was more uniform and finer than that on the powder;
(3) The stress–strain curve of porous titanium obtained by SPS of a Ti powder–fiber mixture consisted of three distinct parts during the compression process: an elastic deformation stage, yield platform stage and densification stage. There was no obvious yield point, and the porous titanium had excellent mechanical properties. The compressive strength of the sample was higher at higher sintering temperatures;
(4) The best sintering process for porous titanium obtained by SPS of a Ti powder–fiber mixture is sintering temperature of 900 °C, and sintering pressure of 20 MPa.
